# Supplementary material for: Univariate and multivariate genomic prediction for agronomic traits in durum wheat under two field conditions
Source: PLoS One. 2024 Nov 14;19(11):e0310886. doi: 10.1371/journal.pone.0310886 (PMC11563401; doi:10.1371/journal.pone.0310886)
Supplement: S1 Table — Raw data were modelled to discern statistical differences among all the factors under examination such as genotype, year, condition, block, genotype by year, genotype by condition, and genotype by year by condition. GY, grain yield; TKW, thousand kernel weight; TW, test weight; GYD, grain yield deviation; NDVI, normalized difference vegetation index; GPC, grain protein content; GPD, grain protein deviation; YI, yellow index; FLA, flag leaf appearance, DTHD, days to heading; DTA, days to anthesis; DTM days to maturity; Gen, Genotype; Cond, Condition. Levels of significance:, <0.001 (***), <0.01 (**), <0.05 (*), non-significant (ns). (DOCX) [file pone.0310886.s001.docx]

| **Trait** | **Gen** | **Year** | **Cond** | **Block** | **Gen*Year** | **Gen*Cond** | **Gen*Year*Cond** |
| --- | --- | --- | --- | --- | --- | --- | --- |
| GY | *** | *** | ns | ** | *** | ns | ns |
| TKW | *** | *** | *** | * | ns | *** | ns |
| TW | *** | *** | *** | *** | *** | *** | *** |
| GYD | *** | *** | *** | *** | *** | ns | *** |
| NDVI | ** | *** | *** | *** | ns | ns | ns |
| GPC | *** | *** | *** | ** | ns | ns | ns |
| GPD | *** | *** | ns | *** | *** | *** | ns |
| YI | *** | *** | *** | *** | *** | ns | ns |
| FLA | *** | *** | *** | *** | *** | *** | *** |
| DTHD | *** | *** | *** | *** | *** | *** | *** |
| DTA | *** | *** | *** | * | *** | *** | *** |
| DTM | *** | *** | *** | *** | *** | *** | *** |

**Table S1.** Analysis of Variance (ANOVA). Raw data were modelled to discern statistical differences among all the factors under examination such as genotype, year, condition, block, genotype by year, genotype by condition, and genotype by year by condition. GY, grain yield; TKW, thousand kernel weight; TW, test weight; GYD, grain yield deviation; NDVI, normalized difference vegetation index; GPC, grain protein content; GPD, grain protein deviation; YI, yellow index; FLA, flag leaf appearance, DTHD, days to heading; DTA, days to anthesis; DTM days to maturity; Gen, Genotype; Cond, Condition. Levels of significance: , <0.001 (***), <0.01 (**), <0.05 (*), non-significant (ns)
