# Supplementary figures and images for: Univariate and multivariate genomic prediction for agronomic traits in durum wheat under two field conditions
Source: PLoS One. 2024 Nov 14;19(11):e0310886. doi: 10.1371/journal.pone.0310886 (PMC11563401; doi:10.1371/journal.pone.0310886)

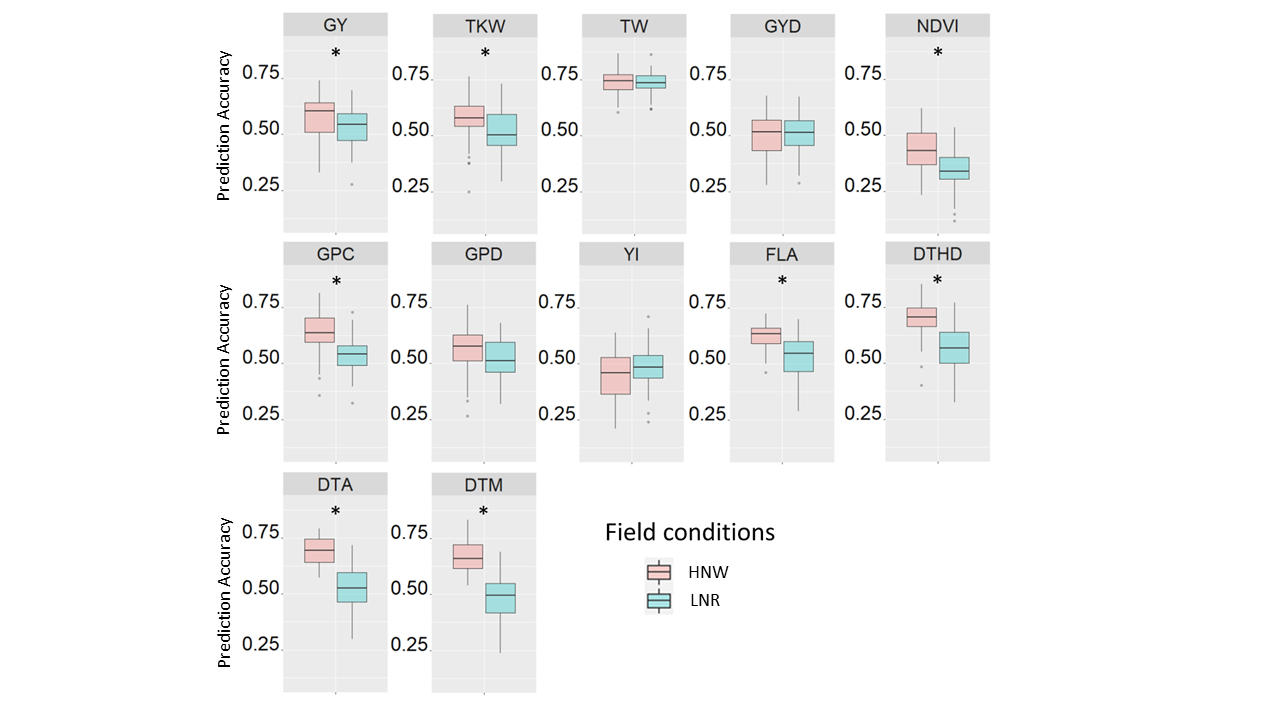

Supplement: S1 Fig — UV-GBLUP model was performed for all traits in both high nitrogen and well-watered (HNW, pink), and (B) low nitrogen and under rainfed (LNR, teal) conditions. The Fisher test was performed to discern statistical differences between the accuracies of two field conditions for the same traits. The statistical significance was indicated by the asterisk. GY, grain yield; TKW, thousand kernel weight; TW, test weight; GYD, grain yield deviation; NDVI, normalized difference vegetation index; GPC, grain protein content; GPD, grain protein deviation; YI, yellow index; FLA, flag leaf appearance, DTHD, days to heading; DTA, days to anthesis; DTM days to maturity. (TIF) [file pone.0310886.s002.tif]
