# Supplementary material for: Univariate and multivariate genomic prediction for agronomic traits in durum wheat under two field conditions
Source: PLoS One. 2024 Nov 14;19(11):e0310886. doi: 10.1371/journal.pone.0310886 (PMC11563401; doi:10.1371/journal.pone.0310886)
Supplement: Attachment — Submitted filename: Response to Reviewers.docx [file pone.0310886.s003.docx]

“Journal requirements:

When submitting your revision, we need you to address these additional requirements.

1. Please ensure that your manuscript meets PLOS ONE's style requirements, including those for file naming. The PLOS ONE style templates can be found at

<https://journals.plos.org/plosone/s/file?id=wjVg/PLOSOne_formatting_sample_main_body.pdf> and

<https://journals.plos.org/plosone/s/file?id=ba62/PLOSOne_formatting_sample_title_authors_affiliations.pdf>”

All the figures and the supporting information are named as requested by the journal.

“2. We note that the grant information you provided in the ‘Funding Information’ and ‘Financial Disclosure’ sections do not match.

When you resubmit, please ensure that you provide the correct grant numbers for the awards you received for your study in the ‘Funding Information’ section.”

We apologize for the oversight. The accurate "funding information" is provided in the attached document, and it has also been appropriately included in the manuscript. “The research was partially funded by European Commission, Grant Agreement number: 727247 — SolACE — H2020-SFS-2016-2017/H2020-SFS-2016-2, and by Italian Ministry of Agriculture, Food and Forestry Policies (MiPAAF), sub-project ‘Tecnologie digitali integrate per il rafforzamento sostenibile di produzioni e trasformazioni agroalimentari (AgroFiliere)’ (AgriDigit programme) (DM 36503.7305.2018 of 20/12/2018).”

“3. We note that you have indicated that data from this study are available upon request. PLOS only allows data to be available upon request if there are legal or ethical restrictions on sharing data publicly. For more information on unacceptable data access restrictions, please see <http://journals.plos.org/plosone/s/data-availability#loc-unacceptable-data-access-restrictions>.

In your revised cover letter, please address the following prompts:

a) If there are ethical or legal restrictions on sharing a de-identified data set, please explain them in detail (e.g., data contain potentially sensitive information, data are owned by a third-party organization, etc.) and who has imposed them (e.g., an ethics committee). Please also provide contact information for a data access committee, ethics committee, or other institutional body to which data requests may be sent.

b) If there are no restrictions, please upload the minimal anonymized data set necessary to replicate your study findings as either Supporting Information files or to a stable, public repository and provide us with the relevant URLs, DOIs, or accession numbers. For a list of acceptable repositories, please see <http://journals.plos.org/plosone/s/data-availability#loc-recommended-repositories>.

We will update your Data Availability statement on your behalf to reflect the information you provide.”

Molecular markers were uploaded in the public repository figshare (https://doi.org/10.6084/m9.figshare.22674667.v1). Similarly, the phenotypic dataset can be obtained by downloading from the public repository Figshare (10.6084/m9.figshare.25554993).

“4. Please include a caption for figure 4.”

The caption of figure 4 was added to the manuscript.

“5. We are unable to open your Supporting Information file [Supporting_information.rar]. Please kindly revise as necessary and re-upload.”

We have regenerated the zip file, now including S1_Fig and S1_Table. We trust that this adjustment will not cause any inconvenience.

“6. Please review your reference list to ensure that it is complete and correct. If you have cited papers that have been retracted, please include the rationale for doing so in the manuscript text, or remove these references and replace them with relevant current references. Any changes to the reference list should be mentioned in the rebuttal letter that accompanies your revised manuscript. If you need to cite a retracted article, indicate the article’s retracted status in the References list and also include a citation and full reference for the retraction notice.”

We changed the reference list in accordance with the new suggestions provided by the reviewers. And any changes is under track mode.

“Reviewer #1: The manuscript by Vitale and coauthors presents an evaluation of a set of genomic prediction models in durum wheat. The manuscript is well written, with well-documented methods, and findings might be useful for the durum wheat breeding community. However, my primary concern lies in the extent of the new information presented in the study as it has been limited to mere model comparison using a training population. It will be great if authors can validate the multivariate models in an independent breeding population, if possible.”

Thank you for your thoughtful feedback. We appreciate your positive remarks regarding the clarity of our manuscript and the documentation of our methods. Regarding your concern about the limited scope of new information, we acknowledge the importance of independent validation in genomic prediction studies. However, we would like to emphasize that cross-validation remains a robust and widely accepted strategy for model testing in wheat breeding. While we understand the value of an independent breeding population for validation, unfortunately, we do not currently have access to another population suitable for this type of validation. Nevertheless, we are committed to refining and extending our study in future works.

“Comments/suggestions for authors to improve the manuscript:
- The usage of terms ‘HIGH’ and ‘LOW’ in reference to the two growing regimes is quite confusing, especially when presenting results for model comparisons. I would suggest the authors replace these terms with another appropriate words.”

We appreciate the reviewer's feedback and acknowledge the confusion caused by the terms 'HIGH' and 'LOW' in our manuscript. To address this concern, we have revised the acronyms for the two field conditions. We now use 'HNW' for high nitrogen and well-watered condition, and 'LNR' for low nitrogen and under rainfed condition.

“-I would request the authors to provide the data along with the R script available so that breeders can actually make use of these findings.”

We recognize the significance of sharing R scripts for the benefit of other researchers and breeders. However, it is important to note that no new scripts were generated for this paper; instead, we utilized functions provided by the packages referenced in the manuscript. To facilitate easy code retrieval for readers, we have incorporated hyperlinks and references to relevant publications (Lines 225-226, 250-251, 265-266) where the scripts employed in this study can be found.

“- The authors estimated the genetic correlation between traits in META-R using equation given in Lines 161-62. Why did authors not use the genomic data (markers’ matrix) for estimating the genetic correlations? I would suggest accounting the markers information for estimating genetic correlation among traits.”

We appreciate the reviewer's insightful comment. While META-R is a robust tool for estimating genetic correlations, we recognize the merit in incorporating molecular marker information for a comprehensive analysis. In response to this suggestion, we have calculated genetic correlations using the markers' information, as detailed in lines 181-189 of the manuscript.

“- Though the methods are well documented, the section presenting methodology for different cross validation schemes lacks clarity. For instance, the authors used the multivariate GP model to predict grain yield using ‘only’ highly correlated traits. Nevertheless, the authors should mention these traits in methods as well as while presenting results.”

We concur with the reviewer's observation regarding the lack of clarity in the "Cross-validation schemes" paragraph. In response, we have made substantial revisions to enhance the clarity of this section in both sub-paragraphs: “Same trait in two field conditions”, and “Grain yield with correlated traits”. Finally, we also added the most genetically correlated traits for both field conditions.

“- In continuation of above, the authors used five correlated traits to predict grain yield. If I am correct, this also included test weight and TKW as covariate in the MT model. In practicality, it would not make sense to use a post-harvest trait to predict grain yield. However, it might be useful in specific scenarios where the data from previous years may be included in models to predict grain yield. The authors need to discuss these scenarios in detail and present the discussion in a way to elucidate how breeders can make use of these models in their programs.”

We appreciate the insightful feedback provided by reviewer #1. The highlighted point is indeed valid, and we acknowledge the significance of this approach, especially in pre-yield trials. In the manuscript, specifically in lines 564-576, we have duly emphasized the importance of this strategy, noting that traits like TKW, TW, and others are observable in early-stage trials, where grain yield might not be readily observed.

“- I would suggest including recent references reporting the evaluation of multivariate GP in wheat.”

We have enriched the manuscript by incorporating additional references pertinent to multivariate GP in wheat (Lines 90-98).

“- The authors used ‘GP pathways’ several times in the manuscript. The word ‘pathway(s)’ does not seem to fit in this context.”

The word “pathway(s)” was removed from the manuscript.

“- Line 20: I believe GP has not been implemented for many years, but more been evaluated.”

We changed it according to the reviewer’s suggestion.

“- Line 325: statically?”

We meant statistically; the error was corrected.

“There are few typos in the manuscript, and I would request the authors to run”

The manuscript was reviewed for addressing typos issue.

“Reviewer #2: Generally, this is an interesting paper and the description of the study mostly understandable and logical. However, the paper has to be improved with major modifications. Also, serious consideration should be given to the English grammar. Although it is understandable, it must be improved.”

We appreciate the positive feedback provided by reviewer #2 on the overall interest and logical coherence of our manuscript. Additionally, we have carefully reviewed the paper, giving due attention to the English grammar concerns highlighted by reviewer #2, and made necessary improvements to enhance the overall quality of the manuscript.

“Comments and suggestions for authors
Title should be modified to “Univariate and Multivariate genomic prediction for agronomic traits in durum wheat under two field conditions”.
Moreover, the manuscript has some deficiencies in its citation practice, particularly in the introduction. The authors need to properly align their statements with relevant and appropriately cited literature, it undermines the credibility and integrity of the work.
The over-use of the pronoun "We” must be avoided in whole paper.”

We appreciate the valuable feedback from the reviewer, and we have implemented the suggested changes to enhance the manuscript. Specifically, the title and introduction have been revised as per the reviewer's recommendations. In response to the concern about the overuse of the pronoun "we," substantial efforts have been made to minimize its usage throughout the paper, aiming for greater clarity and conciseness.

Line 53: delete "is currently becoming" to "becomes" corrected
Line 57: replace "genomic selection" by "GS" corrected
Line 67: Therefore, genomic selection appears …. corrected
Line 69: citation is missing. added
Line 78 and line 80: repetition removed
Line 94: delete "With this in our mind" removed
Line 115-Line 116: on November 28th and December 7^th^ corrected
Line 117: for the first- and second-year trial…. Corrected

“Line 217: please correct the explanation of formulae according to Gill et al (2023)
Gill, H. S., Brar, N., Halder, J., Hall, C., Seabourn, B. W., Chen, Y. R., St. Amand, P., Bernardo, A., Bai, G., Glover, K., Turnipseed, B., & Sehgal, S. K. (2023). Multi-trait genomic selection improves the prediction accuracy of end-use quality traits in hard winter wheat. The Plant Genome, 00, e20331. <https://doi.org/10.1002/tpg2.20331>” We have corrected the explanation of the elements according to the reviewer’s suggestions and according to Sandhu et al., 2022 <https://doi.org/10.3389/fgene.2022.831020>

Table1: please delete unit column and add trait unit in trait column. corrected
Table2: you should add this part “GY, grain yield; TKW, thousand kernel weight; TW, test weight; GYD, grain yield deviation; NDVI, normalized difference vegetation index; GPC, grain protein content; GPD, grain protein deviation; YI, yellow index; FLA, flag leaf appearance, DTHD, days to heading; DTA, days to anthesis; DTM days to maturity.” in table title. added
Delete RF column for MV-CV2. deleted

“Discussion part needs deep improvement: the discussion of results is pretty generic.”

We appreciate the constructive feedback from the reviewer regarding the Discussion section. In response to this valuable input, we made comprehensive improvements to delve deeper into the discussion of results. Specifically, we have expanded our exploration of multivariate genomic prediction applications in the durum wheat breeding program, as also suggested by reviewer #1.

Line 436: Halie et al. corrected
Line 420 to Line 426: this part should be placed in introduction part. The part mentioned by reviewer #2 was correctly replaced in the introduction section.

Line 439 to 442: we cannot compare genomic prediction in maize with that in durum wheat. corrected
Line 449: quality and phenology traits exhibited…. corrected

“Although Conclusions is optional, it should be added in your paper to highlight the paper findings.”

The conclusion was added as suggested by reviewer #2

“In Supplementary Table 1, you should add 3-way interaction "Genotype*Year*Condition"

The 3-way interaction "Genotype*Year*Condition" was added to table S1.
