# Supplementary material for: Univariate and multivariate genomic prediction for agronomic traits in durum wheat under two field conditions
Source: PLoS One. 2024 Nov 14;19(11):e0310886. doi: 10.1371/journal.pone.0310886 (PMC11563401; doi:10.1371/journal.pone.0310886)
Supplement: Attachment — Submitted filename: Response to Reviewers round 2.docx [file pone.0310886.s004.docx]

Dear Sindhu Sareen,

We would like to thank you and the reviewers for your valuable time and insightful comments on our manuscript titled “Univariate and multivariate genomic prediction for agronomic traits in durum wheat under two field conditions.” We appreciate the constructive feedback, which has helped us improve the quality and clarity of our work. In this response letter, we have carefully addressed each of the reviewers’ comments, and we have made the necessary revisions to the manuscript accordingly.

Below, you will find a detailed point-by-point response to the reviewers’ suggestions, along with explanations of the changes made. We believe these revisions have strengthened the manuscript and hope it now meets the expectations of both the reviewers and the journal.

Thank you once again for the opportunity to revise our work, and we look forward to your feedback.

Sincerely,

Paolo Vitale

**Reviewer #1:**

**Comment 1***. About the codes, I am unsure if there would be any issue in creating an online repository and loading the scripts used for analysis. This exercise is important to allow the reproducibility of the results when all other data is being made available. Secondly, the authors provided the links to BGLR and SOMMER repositories. Please note that these resources merely provide information on fitting these models but no information to cross-validate them while using MTGP. I again request the Authors to create a Github (or another repository) and load the codes for usability/reproducibility.*

**Answer 1**: As recommended by the reviewer #1, a GitHub repository containing all the necessary code for running multivariate genomic prediction has been shared. (<https://github.com/paolovitale777/Code-univariate-and-multivariate-genomic-prediction/blob/main/Code>). In the text it was reported in lines 280-281

**Comment 2**: *I still do not see any references to 'recent literature' on multivariate prediction in wheat when there are a lot of recent publications. I believe it authors can use that information to present how breeding programs are using multivariate prediction for a variety of traits in different scenarios.*

**Answer 2**: Thank to reviewer #1 for highlighting this, additional recent examples of multivariate genomic prediction have been added to the introduction (lines 90 to 111) and the discussion (lines 501-502, 556-559).

**Comment 3***: In the updated version, authors have used a matrix notation to represent the GP models. Please make sure the equations are presented appropriately. In this notation, include all terms in bold except the overall mean (u). The same procedure would need to be done for all terms mentioned in the text/paragraph, except for the overall mean and the variance components of the genetic and residual effects.*

**Answer 3:** In response to Reviewer 1's comment, we have followed the convention of using bold typeface for vectors and matrices, while leaving scalar values in regular type. According to this convention, the overall mean and variance components are not bold, as noted by the reviewer. Please consider that overall means $\text{µ}^{\text{'}}$ in equations 8 and 10 represents a vector and for this reason, this term is in bold. If we have overlooked any other terms, please let us know and we will address them promptly.

**Comment 4:** *Please provide the parameters that were used to fit the models and cross-validations. For instance, the burn-ins, iteration, independent runs, etc. Again, providing a code makes it much easier for the reader to go through the modeling method at once.*

**Answer 4:** We are grateful to the reviewer for pointing this out. In response, we have provided the code where all this information can be accessed (see answer 1).

**Comment 5**: *There are several typos in the text and please check the manuscript carefully. For example, GPLUP instead of GBLUP, MN-CV instead of MV-CV, etc.*

**Answer 5:** We carefully reviewed the manuscript to address any typos that might be present in the manuscript.

**Reviewer #2:**

Thus, English grammar revision is well recommended for the full manuscript.

**Comment 1**: *Indeed in the discussion part: the first paragraph should be paraphrased (L571 -> L578).*

**Answer 1:** We are grateful the reviewer #2 for highlighting this. Indeed, we improved the clarity and the readability of the first paragraph of the discussion.

**Comment 2**: *The use of pronoun "We" should be avoided.*

**Answer 2**: The use of the pronoun "We" has been drastically reduced.
